# A 17-year trend analysis of malaria at Adi Arkay, north Gondar zone, Northwest Ethiopia

**DOI:** 10.1186/s12936-018-2310-9

**Published:** 2018-04-06

**Authors:** Habtie Tesfa, Abebe Genetu Bayih, Ayalew Jejaw Zeleke

**Affiliations:** Department of Medical Parasitology, School of Biomedical and Laboratory Sciences, College of Medicine and Health Sciences, University of Gondar, Gondar, Ethiopia

**Keywords:** Adi Arkay, Malaria, Northwest Ethiopia, Trend

## Abstract

**Background:**

Malaria is one of the leading causes of death worldwide. This study aimed to determine the trend of malaria among febrile patients seeking treatment over 17 year (1997–2013) at Adi Arkay, Northwest Ethiopia.

**Methods:**

A 17-year malaria microscopy data were extracted retrospectively at Adi Arkay health centre. Time series and curve estimation analysis were used to evaluate trends in the data. Pearson’s Chi square test was also used to describe associations of variables.

**Results:**

Over 17 years, 20,483 blood films were requested for malaria diagnosis at the health centre. Out of this, 7428 (36.1%) were microscopically confirmed malaria cases. *Plasmodium falciparum, Plasmodium vivax,* and their mixed infection accounted for 68.85, 28.79, and 2.34% of all malaria cases, respectively. There was a remarkable reduction of overall malaria during the 17 years. Malaria was reported in all age groups of both sexes, but its positivity rate was significantly higher in males and in the 15–24 years than their counterparts.

**Conclusion:**

In relative terms, the overall positivity rate of malaria in the area over 17 years showed a significant reduction, but its magnitude as a public health problem is still alarming. *Plasmodium falciparum* played a significant role in the remarkable drop of overall malaria in the area, whereas vivax malaria remained unchanged. Therefore, control measures should continue to strengthen targeting both predominant malaria parasites in the area.

## Background

Malaria is one of the leading causes of death in the world. According to WHO report, there were 214 million cases and 438,000 deaths that were attributed to malaria in 2015 [1]. It is a deadly disease, especially in Africa, including Ethiopia [2], where the disease is prevalent at altitudes below 2000 m above sea level (asl). Its peak transmission occurs from September to December and April to May, coinciding with the major agricultural seasons. This has a negative impact on the Ethiopian subsistence agriculture-based economy [2]. Areas below 2000 m asl cover three-quarters of the country’s land mass. Malaria epidemics occur every 5–8 years in these parts of the country [3].

In Ethiopia, *Plasmodium falciparum* and *Plasmodium vivax* account for 60 and 40% of the total reported cases, respectively. These *Plasmodium* species substantially contribute to malaria morbidity in the country [4]. However, information on the trend of *P. falciparum* and *P. vivax* malaria in endemic areas is still insufficient.

Several countries have eliminated malaria since a programme for its global eradication was launched in 1955. However, no country has declared malaria elimination in Africa. The Global Technical Strategy for Malaria (GTS) calls for the elimination of malaria in at least 10, 20, and 35 countries by 2020, 2025, and 2035, respectively [5]. In fact, the fight against malaria has shown a remarkable progress in controlling the disease over the last 20 years in Ethiopia. The burden has declined significantly, over the last decade, due to an improved coverage of high impact interventions, including the distribution of insecticide-treated nets (ITNs) and indoor residual spraying (IRS). Moreover, prompt and effective treatment with artemisinin-based combination therapy (ACT) was introduced to all health facilities free of charge to all age groups as a first line treatment for uncomplicated malaria in 2004. Targeted areas for key malaria control interventions were selected in accordance with criteria such as altitude (< 2000 m), morbidity data, and history of epidemics [6–8]. Accordingly, Adi Arkay was one of the hot spot areas where malaria epidemic control interventions were carried out. Epidemiological data such as trends of malaria positivity rates at health institution is essential to design appropriate interventions. Furthermore, there is no published data regarding the trends of malaria cases at health institution and particularly at Adi Arkay town. Therefore, the present study aims at assessing the malaria positivity rate for the past 17 years at the institution as a proxy measure for the trend of malaria in the area which may contribute to evidence-based decision on malaria control activities.

## Methods

### Study area and study population

The study was conducted at Adi Arkay health centre. Adi Arkay is one of the Woredas (administrative districts) in Amhara region in Ethiopia. It is situated on the northern slopes of *Ras Dashen*, which is one of the highest peaks in Africa. It is 907 km north of Addis Ababa (the capital city of Ethiopia), and 180 km north of the ancient city of Gondar [9]. This district is malarious and with a total population of about 93,763. The altitude of the district ranges between 1750 and 2100 m above sea level. The majority of the population depends on subsistence farming and livestock breeding. Malaria is the most prevalent seasonal disease in the area, accounted as second of all the reported diseases in the health center. Adi Arkay Health Centre was founded 20 years ago. Unlike most malaria endemic areas of the country, there are no immigrant laborers in the area and this health facility is used by the local population and has a very high patient load. The study population in this study were all malaria suspected patients (including both sexes and any age groups) who were visiting the health centre for the past 17 years.

### Study design

A retrospective study was conducted to determine the 17 years trend of malaria by reviewing blood film malaria reports at Adi Arkay health centre.

### Data collection

Seventeen years (1997–2013) data regarding malaria were extracted from Adi Arkay heath centre. Data on both negative and positive microscopically confirmed malaria suspected cases were included in the study. In Ethiopia, microscopy is the major tool used to diagnose malaria. In this health centre, a well-prepared Giemsa-stained blood film (both thick and thin smear) was used to diagnose malaria in the laboratory [10]. Information regarding the patient’s age, sex, month, and year of examination were collected using the check list established for this study. Unfortunately, complete (formal) data regarding major interventions done against malaria and other environmental factors were not collected.

### Data analysis

Data were extracted from laboratory log books and summarized using Microsoft Excel. Then, data was entered and analyzed using SPSS 20 software package. Sequence chart and curve estimation analysis were used to evaluate trends of the data. Pearson’s Chi square test was used to describe association of variables.

## Results

### Annual trends of malaria prevalence in Adi Arkay health centre

Over a period of 17 years (1997–2013), 20,483 blood films were requested for malaria diagnosis at Adi Arkay health centre of which 7392 (36.1%) were microscopically confirmed malaria cases. There were significant fluctuations and reduction trends of overall malaria during the past 17 years, with a maximum of 51.5% and a minimum of 26.6% of cases in 2001 and 2009, respectively. Although a remarkable reduction of all types of malaria was observed, a high predominance of *P. falciparum* was reported over *P. vivax* in each year. *Plasmodium falciparum, P. vivax*, and their mixed infection accounted for 68.85, 28.79, and 2.34% of the malaria cases, respectively. A minimum of 13.8% and a maximum of 39.3% malaria cases due to *P. falciparum* were reported in 2009 and 2001, respectively. In most years, the remarkable decrease in the proportion of malaria infected cases was almost all due to *P. falciparum.* In contrast, the fluctuation and reduction of malaria due to *P. vivax* was not significant. The least (7.9%) and the highest (12.9%) peaks of malaria cases due to vivax malaria were observed in 2010 and 1998, respectively. Moreover, mixed infection did not show a significant fluctuating trend in the area in the 17 years (Table 1).

**Table 1:** Annual trends of malaria positivity rates at Adi Arkay health centre from 1997 to 2013, Adi Arkay Town, Ethiopia

Year	Total no. of blood films examined	Total no. of malaria cases (%)	*P. falciparum* cases (%)	*P. vivax* cases (%)	Mixed (%)
1997	380	180 (47.4)	124 (32.6)	48 (12.6)	8 (2.1)
1998	695	326 (46.9)	267 (38.4)	55 (7.9)	4 (0.6)
1999	626	299 (47.8)	231 (36.9)	62 (9.9)	6 (1)
2000	230	105 (45.6)	77 (33.3)	28 (12.1)	0 (0.2)
2001	811	418 (51.5)	319 (39.3)	93 (11.5)	6 (0.8)
2002	1495	665 (44.5)	520 (34.8)	135 (9.0)	10 (0.7)
2003	1272	563 (44.3)	432 (34)	117 (9.2)	13 (1)
2004	618	260 (42)	184 (29.8)	70 (11.3)	6 (0.9)
2005	2592	980 (37.8)	728 (28.1)	251 (9.7)	0 (0)
2006	1665	636 (38.2)	463 (27.8)	160 (9.6)	13 (0.8)
2007	914	314 (34.4)	211 (23.1)	101 (11.1)	2 (0.2)
2008	979	319 (32.6)	234 (23.9)	85 (8.7)	0 (0)
2009	369	98 (26.6)	51 (13.8)	47 (12.7)	0 (0)
2010	743	222 (29.9)	122 (16.4)	96 (12.9)	4 (0.5)
2011	1458	424 (29.1)	286 (19.6)	136 (9.3)	1 (0.1)
2012	2155	640 (29.7)	356 (16.5)	254 (11.8)	30 (1.4)
2013	3481	943 (27.1)	484 (13.9)	390 (11.2)	70 (2)
Total	20,483	7392 (36.1%)	5089 (24.8)	2128 (10.4)	173 (0.8)

**Fig. 1:**
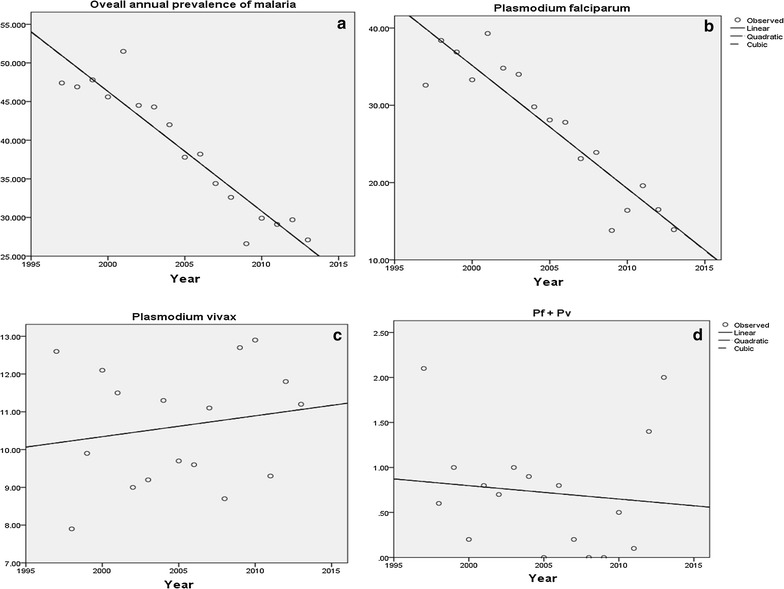
Curve estimation models for malaria investigation in years in Adi Arkay health centre from 1997 to 2013, Adi Arkay Town, Ethiopia

A curve estimation analysis done using three models, namely linear, quadratic and cubic also showed a statistically significant reduction of overall malaria positivity rates from time to time (each of the models has R square = 0.85, P < 0.001) (Fig. 1a). The same result was observed for malaria due to *P. falciparum* showing a significant reduction in the area during the 17 years (each of the models has R square = 0.89, *P *< 0.001) (Fig. 1b). During the 17 years, as far as these models were used, *P. vivax* did not show a significant reduction (each of the models has R square = 0.03, *P *= 0.492), even showing a slight increase across the years (Fig. 1c). Similarly, mixed infection did not show a significant reduction across the 17 years according to analysis done using the models (each of the models has R square = 0.01, *P *= 0.20) (Fig. 1d).

### Seasonal variations of malaria prevalence at Adi Arkay health centre

**Fig. 2:**
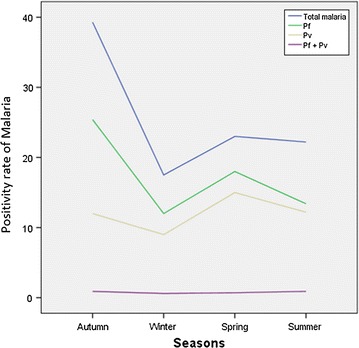
Distribution of Plasmodium in different seasons in Adi Arkay health centre from 1997 to 2013, Adi Arkay Town, Ethiopia

Despite the apparent fluctuation of total malaria trends over 17 years in the study area, malaria cases occurred throughout the year. However, there was a significant seasonal variation (*P *= 0.04). The highest peak of total malaria positivity rate was observed during autumn, i.e. the months of September, October and November (just after the major rainy season) and the minimum positivity rate was observed during winter (the dry season in the months of December, January and February). Similarly, the maximum and minimum positivity rate of *P. falciparum* followed similar patterns. On the other hand, the positivity rate of malaria due to *P. vivax* was the highest during April and May (spring, short rainy season). Malaria cases due to mixed infections were almost similar in all seasons (Fig. 2).

### Prevalence of malaria in relation to age and sex at Adi Arkay health centre

**Fig. 3:**
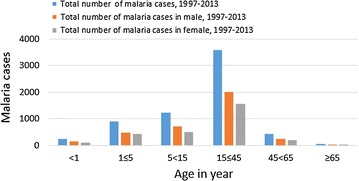
Malaria cases by sex and age in Adi Arkay health centre from 1997 to 2013, Adi Arkay Town, Ethiopia

According to the record review over the 17 years in the study area, malaria was reported in all age groups, but the 15–45 years age group was more affected (3521 of the total 7392 malaria cases), followed by the 5–15 years group (1211 of 7392 malaria cases). In all age groups, males were more affected than females, and the difference was statistically significant (*P *< 0.001) (Fig. 3).

**Fig. 4:**
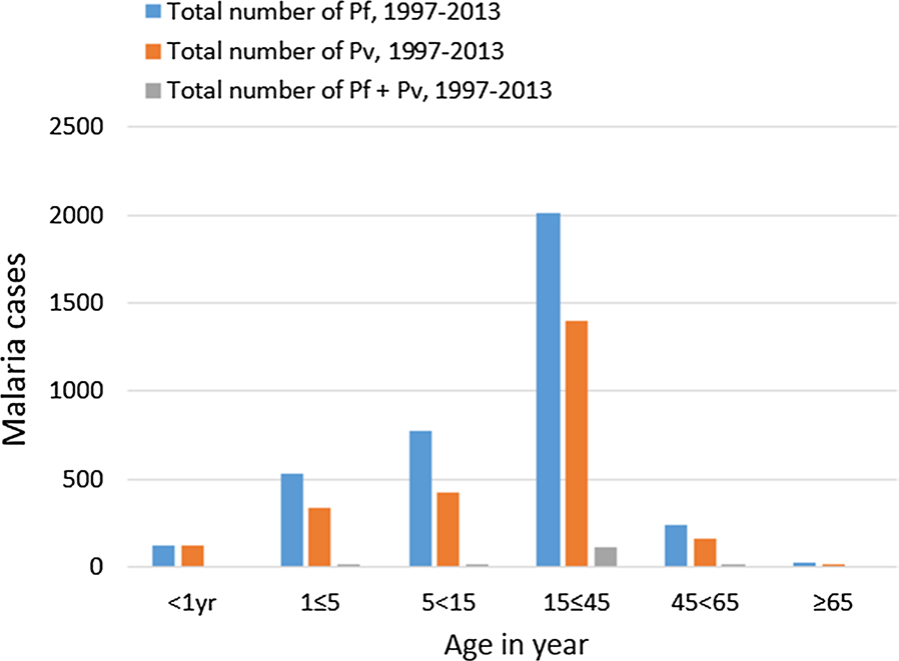
Distribution of *Plasmodium* species by age in Adi Arkay health center from 1997 to 2013, Adi Arkay Town, Ethiopia

The comparison of the *Plasmodium* species and age groups in the study area showed that *P. falciparum* was the predominant parasite in all age groups, but higher in the 15–45 years of age group than in other groups. Similarly, the same age group (15–45 years) was the most affected by *P. vivax,* and mixed infections which were the least prevalent in all age groups (Fig. 4).

## Discussion

Over the 17 years (1997–2013), a 36.1% annual mean positivity rate of malaria was reported in the study area. This is lower than that of a study conducted at Kola Diba health centre [6]. However, the result of this study indicates that malaria was a significant health burden in the area. Their differences might be related to variations in malaria diagnosis techniques and the skills of the laboratory personnel to detect and identify malaria parasites. Moreover, the implementation of malaria prevention and control activities might differ from one area to another showing that the interventions in this community might have been stronger. This study also revealed that malaria cases due to *P. falciparum* and *P. vivax* species accounted for 68.85 and 28.79% of the cases, respectively. The remaining 2.34% of malaria positivity rate was due to mixed infections. This is not in line with the national malaria parasite distribution figure in Ethiopia, which showed that *P. falciparum* and *P. vivax* accounting for 60 and 40% of the cases, respectively [6, 11]. The national estimation of malaria parasites indicates the average distribution in the whole country, whereas the present study is limited to only a small part of Ethiopia could have resulted in the variation of the prevalence.

Over the 17 years, there was a dropping trend of total malaria cases from 47.4% in 1997 to 26.6% in 2009 (Table 1). The curve estimation analysis models (linear, quadratic and cubic) which explained practically significant overall malaria trend reduction across the years were used (each models showed; R^2^ = 0.85, P < 0.001). This overall reduction of malaria is mainly related to the dramatic drop in the rate of falciparum malaria. This declining trend testifies the commitment of the government and other partners in Ethiopia. Malaria control has been one of the major components of the country’s prioritized health sector agenda since 2004. The Ethiopian Government together with collaborators launched multiple interventions for malaria prevention and control for the entire communities at risk including the study area [7]. The national strategic malaria elimination plan currently recommends key intervention methods including prompt diagnosis using rapid diagnostic tests (RDT), artemisinin combination therapy (ACT) as first line drug to treat uncomplicated *Plasmodium falciparum* malaria, and targeting the vector using indoor residual spraying (IRS) and long lasting insecticide treated nets (LLITNs). Chemoprophylaxis and environmental management are also key intervention measures applied to control malaria [7, 12, 13]. Moreover, in the past several years, malaria control and prevention activities were intensified by all stockholders in the area. Awareness creation of the community about malaria transmission and control methods, the increment of budget and increased accessibility of ITNs to the community were the major interventions made by health professionals and representative of the government working in the town. All these efforts might have resulted in such significant reduction of malaria positivity rate.

Despite the national malaria intervention initiatives [7], the prevalence of *P. vivax* did not drop, instead showed a slight rise (Fig. 1). Its persistence may be related to the emergence of chloroquine resistance in *P. vivax* [11, 14]. Moreover, its ability to assume a dormant stage in the liver during its life-cycle might be the other reason for the insignificant reduction. A control strategy on the distribution of Co Artem^®^ in endemic areas targets mostly *P. falciparum* and a greater emphasis on *P. vivax* is needed [7]. The month of September, October and November is the season when the highest peak of malaria has been frequently reported from different parts of Ethiopia [6, 15, 16]. In a similar fashion, over the study period, the highest prevalence of malaria was observed during this season followed by spring (March, April, and May) in the study area during the study period. However, malaria cases were reported in the area in all seasons across the 17 years.

The present study also revealed a slightly higher positivity rate of malaria among males (39.4%) than females (32.4%). This is in agreement with the report from other parts of Ethiopia [6]. Most people in Adi Akay are farmers, thus, most of the time males are engaged in agricultural activities during which they are more exposed to the bite of Anopheles mosquitoes, vectors of malaria parasites. The 15–45 year age group was the most affected age groups in both sexes (Fig. 4), and similar findings were also reported from Kola Diba, Ethiopia [6]. This is the most productive age group which actively involves in several agricultural activities which expose them to the infection.

The present study indicates that a 17 years cases of malaria at the health institution as a proxy measure in the trend of malaria, but secondary data was used for analysis and this might have affected the accuracy of positivity rate of malaria. Thus, it has a limitation and should be interpreted with caution.

## Conclusion

There has been a significant declining trend of the overall positivity rate of malaria in the study area. However, it is still alarming and requires extra efforts for further reduction. The declining trend of the overall positivity rate was mainly due to the remarkable decrease of *P. falciparum* rather than *P. vivax* infection. The implementation of malaria control activities should be reinforced, taking into consideration the variables identified in this study.

## Abbreviations

ACT: artemisinin-based combination therapy
HEWs: health extension workers
IRS: indoor residual spray
LLITNs: long-lasting-insecticide-treated nets
ITNs: insecticide-treated nets
RDT: rapid diagnostic tests

## References

[1] WHO (2015). World health statistics 2015.

[2] Ayele DG, Zewotir TT, Mwambi HG (2012). Prevalence and risk factors of malaria in Ethiopia. Malar J..

[3] Jima D, Getachew A, Bilak H, Steketee RW, Emerson PM, Graves PM, Gebre T, Reithinger R, Hwang J (2010). Malaria indicator survey 2007, Ethiopia: coverage and use of major malaria prevention and control interventions. Malar J..

[4] Seyoum D, Kifle YG, Rondeau V, Yewhalaw D, Duchateau L, Rosas-Aguirre A (2016). Identification of different malaria patterns due to *Plasmodium falciparum* and *Plasmodium vivax* in Ethiopian children: a prospective cohort study. Malar J..

[5] WHO (2016). Eliminating malaria.

[6] Alemu A, Muluye D, Mihret M, Adugna M, Gebeyaw M (2012). Ten year trend analysis of malaria prevalence in Kola Diba, North Gondar, Northwest Ethiopia. Parasit Vectors..

[7] Aregawi M, Lynch M, Bekele W, Kebede H, Jima D, Taffese HS (2014). Time series analysis of trends in malaria cases and deaths at hospitals and the effect of antimalarial interventions, 2001–2011, Ethiopia. PLoS ONE..

[8] Abeku TA, Helinski ME, Kirby MJ, Kefyalew T, Awano T, Batisso E (2015). Monitoring changes in malaria epidemiology and effectiveness of interventions in Ethiopia and Uganda: beyond Garki Project baseline survey. Malar J..

[9] Guinand Y, Ugas M. Underdeveloped, drought prone, food insecure: reflections on living conditions in parts of the Semen Mountains. Assess Mission; 1999. p. 18–29.

[10] WHO (2010). Basic Malaria Microscopy: Tutor’s guide.

[11] Golassa L, Erko B, Baliraine FN, Aseffa A, Swedberg G (2015). Polymorphisms in chloroquine resistance-associated genes in *Plasmodium vivax* in Ethiopia. Malar J..

[12] Ababa A (2003). Postnatal care.

[13] Keiser J, Singer BH, Utzinger J (2005). Reducing the burden of malaria in different eco-epidemiological settings with environmental management: a systematic review. Lancet Infect Dis..

[14] Price RN, von Seidlein L, Valecha N, Nosten F, Baird JK, White NJ (2014). Global extent of chloroquine-resistant *Plasmodium vivax*: a systematic review and meta-analysis. Lancet Infect Dis..

[15] Alemu A, Abebe G, Tsegaye W, Golassa L (2011). Climatic variables and malaria transmission dynamics in Jimma town, South West Ethiopia. Parasit Vectors..

[16] Sena LD, Deressa WA, Ali AA (2014). Analysis of trend of malaria prevalence in South-West Ethiopia: a retrospective comparative study. Malar J..

